# Maltoma veiled in the lung-a rare case of pulmonary and gastric maltoma

**DOI:** 10.1016/j.rmcr.2021.101403

**Published:** 2021-03-19

**Authors:** Adeel Nasrullah, Ayla Gordon, Anam Javed, Usman Tariq, Atif Raja, Ahmad Alhajhusain

**Affiliations:** a Department of Medicine, Allegheny Health Network; Pittsburgh, PA, USA; b Departement of Pulmonology and Critical Care, Allegheny Health Network, Pittsburgh, PA, USA; c Department of Pathology, Allegheny Health Network, Pittsburgh, PA, USA

**Keywords:** Pulmonary maltoma, Lymphoma, Extranodal marginal zone lymphoma

## Abstract

Mucosa associated lymphoid tissue (MALT) is a type of B-cell lymphoma that is commonly observed in the gastrointestinal site, most frequently occurring in the stomach. However, the incidence of this type of lymphoma in the respiratory tract is very uncommon. We report a case of this rare clinical entity in a patient who presented with non-symptomatology and was diagnosed with pulmonary MALT lymphoma (pMALToma).

## 1 Introduction

Mucosa-associated lymphoid tissue (MALT) lymphoma is an extra-nodal low-grade marginal zone B-cell lymphoma. The condition is commonly associated with autoimmune diseases, in particular, autoimmune thyroiditis and Sjogren's syndrome [2], as well as other chronic inflammatory conditions and site-specific protracted infections [1]. Implicated microorganisms include *Helicobacter pylori, Borrelia burgdorferi, Chlamydia psittaci, and Mycobacterium tuberculosis; associated with* gastric MALT lymphoma, B-cell cutaneous lymphoma, ocular adnexal lymphoma, and pulmonary MALT lymphoma, respectively [1]. While the exact pathology of MALT lymphoma remains unknown, the persistent antigenic exposure and additional oncologic events in extra-nodal tissues are attributed to monoclonal lymphoid hyperplasia and subsequent extranodal lymphoma [3]. Most commonly involved sites are the stomach followed by the bowel, thyroid gland, bladder, skin, and salivary glands [4]. Herein, we present a rare case of pulmonary MALT lymphoma (pMALToma) masking as chronic sarcoidosis. We will further discuss the condition's differing and often vague symptomology, varying diagnostic approaches, and treatment modalities used to curb this rare ailment.

## 2 Case presentation

A 59-year-old African American female with a history of type 2 diabetes mellitus (DM) and chronic gastroesophageal reflux disease (GERD) was evaluated in the pulmonology clinic with complaints of persistent dyspnea on exertion (DOE) and cough persistent for six months. She also cited intermittent, generalized abdominal pain and a twelve-pound weight loss in the previous four months. On further inquiry, she reported that she had undergone computed tomography (CT) scan of her chest six years earlier, which showed multiple bilateral nodules. Owing to her refusal, a transbronchial biopsy could not be performed, and her preliminary diagnosis of sarcoidosis was upheld. The patient was a lifelong non-smoker and denied a family history of lung diseases.

**Fig. 1:**
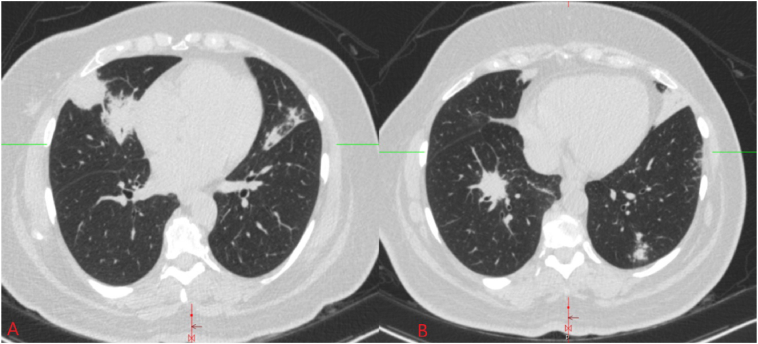
Multiple nodular opacities are present bilaterally in lungs.

**Fig. 2:**
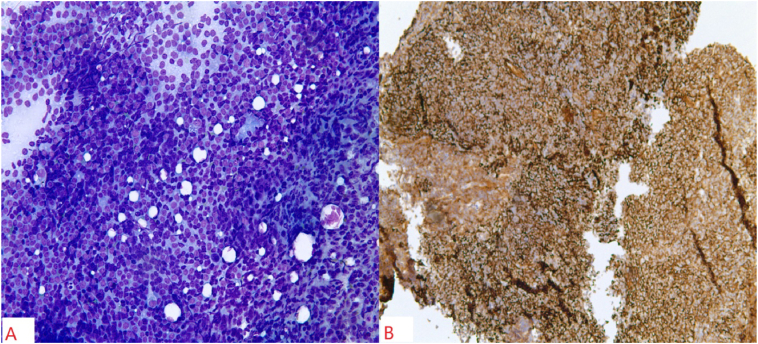
Section A shows dense, monotonous population of centrocyte-like cells while part B demonstrates positivity for CD 21.

On clinical exam, the patient was hemodynamically stable with equal air entry bilaterally. Initial laboratory workup including complete blood count and complete metabolic picture were unremarkable. A repeat chest CT scan was obtained, which revealed nodular areas of consolidation in both lungs (Fig. 1). Due to her apprehensions against general anesthesia, a CT-guided right lung biopsy was performed, which disclosed a dense, monotonous population of centrocyte-like cells (Fig. 2). Immunohistochemistry stained positive for CD20, Bcl-2, and CD21 while *in situ* hybridization (FISH) showed kappa light-chain restriction, which confirmed the diagnosis of a marginal zone lymphoma (MZL) (Fig. 2).

**Fig. 3:**
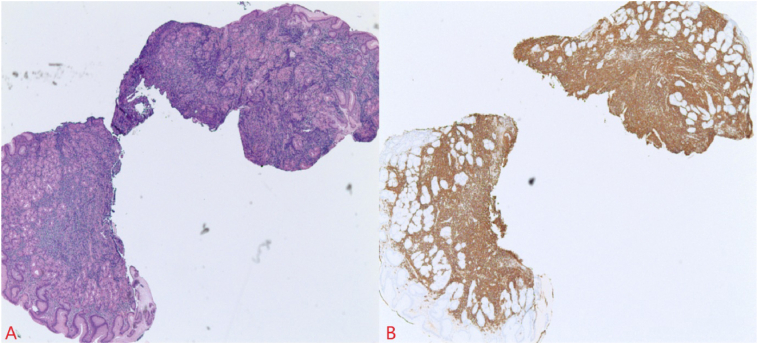
Section A shows mono-morphos small cell lymphoid infiltrate with slightly irregular nuclear contours and moderate pale cytoplasm (centrocyte-like morphology) infiltrating the lamina propria with focal infiltration of the epithelial structures while section B demonstrates CD20 diffusely positive in a monotonous population of B-cells.

Subsequent staging investigations included negative bone marrow biopsy and an esophagogastroduodenoscopy (EGD). Gastric biopsies revealed an atypical B-cell lymphoid infiltrate consistent with an extra-nodal marginal zone lymphoma of MALT (Fig. 3). Additionally, *Helicobacter pylori* stool antigen and immunohistochemistry were negative. The gastric mucosa was involved by a monomorphous small cell lymphoid infiltrate with slightly irregular nuclear contours and moderate pale cytoplasm (centrocyte-like morphology) infiltrating the lamina propria with focal infiltration of the epithelial structures. Immunohistochemistry revealed positivity for CD 20 and a low proliferation index (Ki67).

In the ensuing week after staging, the patient was admitted to the inpatient setting with complaints of excessive abdominal pain, nausea, and vomiting. A CT scan of the abdomen and pelvis unveiled a hypo-dense 2.2 cm pancreatic neck. An endoscopic ultrasound (EUS) with biopsy established a new diagnosis of pancreatic ductal adenocarcinoma.

Medical oncology was consulted for further management, and the patient was started on Gemcitabine and Abraxane, with an outpatient follow-up on a regular basis.

## 3 Discussion

Pulmonary non-Hodgkin's lymphoma (NHL) is an infrequent entity with an annual incidence of 1:313000 [5]. It comprises <1% of all NHLs, with marginal zone lymphomas (MZL) of MALT variety constituting more than two-thirds of its morphological presentation [6]. It is unrelated to smoking and is equally prevalent in males and females [7]. The median age of diagnosis is during the fifth decade of life [8]. Chronic antigenic presence and resultant extended immune response are attributed to its causality [3].

Pulmonary MALT lymphoma (pMALToma) is a challenging diagnosis due to the indolent nature of the disease, coupled with its non-specific symptomology and radiological findings [9]. A retrospective study surveying patients with pMALToma across fifteen years determined that 36% (n = 63) were asymptomatic at the time of diagnosis while other patients reported non-discrete symptoms such as a dry cough, dyspnea on exertion, chest pain, or hemoptysis. A minority of symptomatic patients also reported B-type symptoms such as fever, night sweats, and weight loss [8]. The presence of these vague symptoms can lead to misdiagnoses such as viral or bacterial pneumonia, cancer, sarcoidosis, or tuberculosis in endemic regions. Clinical examination is generally unremarkable and makes a scarce contribution to the final diagnosis [10]. Diagnostic workup consists of laboratory investigations and radiologic imaging. Blood counts can display cytopenia, and a resultant increased cell turnover can lead to elevated LDH levels. However, positron emission tomography (PET) scan has limited utility in the diagnosis of pMALToma as these tumors are slow-growing and have reduced uptake of fluorodeoxyglucose (FDG); hence, its use is controversial [11]. Radiologic findings can vary with commonly seen patterns, as summarized in Table 1 [5,7].

**Table 1:** Prevalence of different radiological presentations in Pulmonary Maltoma.

	Findings	Prevalence (percentage)
1	Air bronchograms	50%
2	Nodular lesions	55%
3	Lung mass	50%
4	Ground glass opacities	25%
5	Consolidations	55%
6	Micronodules	20%
7	Septal lines	10%
8	Hilar lymphadenopathy	15%
9	Pleural effusion	10%

A definitive diagnosis of a pMALToma can be acquired via a histological analysis of a biopsied tissue specimen [12]. A lung biopsy can be obtained through a variety of approaches including transbronchial biopsy, radiologically guided transthoracic core needle-biopsy, video-assisted thoracoscopic surgery, or thoracotomy-based approaches depending on the lesion site and patient profile. Bronchoscopy has greater utility in polypoidal endobronchial lesions, and bronchoalveolar lavage can further aid in deciphering underlying pathology based on cytology results [7,13]. Transbronchial biopsy has a sensitivity of 31% and specificity of 88% in diagnosing pMALToma [7]. If a transbronchial biopsy is not possible, then a CT-guided transthoracic aspiration and biopsy are indicated as was performed in our patient. This diagnostic modality has a sensitivity of 80% for pMALToma. At times, however, a tissue sample may be deficient, especially in patients with an uncharacteristic exhibition on a chest CT, in which case a surgical biopsy could be used as a basis for both diagnosis and therapeutic intervention [7,12].

A standard tissue diagnosis requires Hematoxylin and Eosin (H&E) staining, followed by immunochemical testing and molecular genetic analysis. Monoclonal B cells display positive cell markers for CD20, CD21, and Bcl2 but are negative for IgD, cyclin-D1, Bcl6, CD10, and CD5, which excludes other common lymphoepithelial lesions [5]. Immunohistochemistry of right lung mass of our patient revealed positivity for CD20, Bcl2 and CD21, and negativity for Cyclin D1 and CD10. Monoclonal B- cells have an increased tendency to produce monoclonal immunoglobulins due to persistent antigenic stimulation [8]. Restricted immunoglobulin production differentiates reactive lymphatic lesions from monoclonal lymphoid hyperplasia. MALT lymphomas typically have antigenically driven somatic mutations in the immunoglobulin heavy-chain variable region (IGHV), which can be seen on IGHV sequence analysis. IGHV3 and IGHV4 are consistently seen in pulmonary lymphomas [14]. The most common cytogenetic abnormalities are translocations, t(11; 18) (q21; q21) being the most commonly found translocation in MALT lymphomas. Reverse transcriptase or fluorescence *in situ* hybridization (FISH) can detect the disease in 42% of pulmonary cases [7]. If needed, bone marrow biopsy can be performed, which yields positive in 13–30% of cases [15].

Choice of treatment is multifactorial and based on symptomology, site, staging of disease, and associated comorbidities. Due to the multifocality of the tumor, extrapulmonary MALToma should be investigated by CT abdomen and pelvis, and upper gastrointestinal endoscopy should be performed as was done in the present case. In more than 25% of cases, synchronous extra-nodal MZL is found [7]. Although there is no single recommended treatment strategy, common approaches are wait-and-watch, surgical excision, chemotherapy, and radiotherapy. Asymptomatic patients can be managed with a wait-and-watch approach, but one third of patients have progressive disease and may require further intervention [16].

Interestingly, while MALT lymphomas are associated with chronic inflammatory states, even in the absence of active infection, a 14-day course of therapy with clarithromycin 2 g/day has led to tumor size reduction in several cases [17]. Surgical treatment is reasonable in cases where the lesion is easily accessible and completely resectable. In cases with multiple lesions, as seen in our patient, chemotherapy and radiotherapy are the main approaches to treatment. Chemotherapy with rituximab-chlorambucil is considered first-line as the combination has shown a 5-year survival rate in 68% of patients [18]. Other viable regimens include fludarabine and bortezomib [19]. Due to findings of a pancreatic malignancy in our patient, the chosen chemotherapy regimen was aimed at the pancreatic cancer, with simultaneous activity against pMALToma.

pMALToma generally has a favorable prognosis due to the prospect of spontaneous regression. The survival rate has been reported to be 91%, 59%, and 53% at one year, five years, and ten years respectively [17,20]. Advanced histopathological stage and presence of amyloid in tumor confer a poor prognosis, as does age greater than 70 years, advanced stage IV disease, and elevated beta-2 microglobulin [21]. Despite adequate treatment, prolonged follow up is usually deemed necessary due to the risk of recurrence in more than 50% of cases [7].

## 4 Conclusions

The rarity of pMALToma, coupled with its vague symptomatology and resemblance to other chronic pulmonary conditions, such as sarcoidosis, can delay diagnosis and hinder the application of appropriate treatment modalities. Histological evaluation remains the cornerstone of establishing causality in suggestive presentations. Treatment of chronic underlying infections and chemotherapeutic interventions remain the mainstay in the management of this rare lymphoma-subtype with favorable survival rates when promptly addressed.

## Prior presentations

Poster presentationa at American Thoracic Society Conference 2020.

## Funding

This research did not receive any specific grant from funding agencies in the public, commercial, or not-for-profit sectors.

## Declaration of competing interest

The authors report no conflicts of interest.
